# Investigation of systemic immune-inflammation index, neutrophil/high-density lipoprotein ratio, lymphocyte/high-density lipoprotein ratio, and monocyte/high-density lipoprotein ratio as indicators of inflammation in patients with schizophrenia and bipolar disorder

**DOI:** 10.3389/fpsyt.2022.941728

**Published:** 2022-07-26

**Authors:** Yanyan Wei, Tingting Wang, Guoguang Li, Junhui Feng, Lianbang Deng, Haiting Xu, Lu Yin, Jinbao Ma, Dongning Chen, Jingxu Chen

**Affiliations:** ^1^Beijing Hui-Long-Guan Hospital, Peking University, Beijing, China; ^2^School of Mental Health, Bengbu Medical College, Bengbu, Anhui, China; ^3^The Fourth People’s Hospital of Liaocheng, Liaocheng, Shandong, China; ^4^Jining Psychiatric Hospital, Jining, Shandong, China; ^5^Beijing Tongren Hospital, Beijing, China

**Keywords:** schizophrenia, bipolar disorder, inflammation, lipid metabolism, MHR

## Abstract

**Background:**

The systemic immune-inflammation index (SII), system inflammation response index (SIRI), neutrophil/high-density lipoprotein (HDL) ratio (NHR), lymphocyte/HDL ratio (LHR), monocyte/HDL ratio (MHR), and platelet/HDL ratio (PHR) have been recently investigated as new markers for inflammation. The purpose of this research is to use large-scale clinical data to discuss and compare the predictive ability of the SII, SIRI, NHR, LHR, MHR, and PHR in patients with schizophrenia (SCZ) and bipolar disorder (BD), to investigate potential biomarkers.

**Materials and methods:**

In this retrospective, naturalistic, cross-sectional study, we collected the hematological parameter data of 13,329 patients with SCZ, 4,061 patients with BD manic episodes (BD-M), and 1,944 patients with BD depressive episodes (BD-D), and 5,810 healthy subjects served as the healthy control (HC) group. The differences in the SII, SIRI, NHR, LHR, MHR, and PHR were analyzed, and a receiver operating characteristic (ROC) curve was used to analyze the diagnostic potential of these parameters.

**Results:**

Compared with the HC group, the values of the SII, SIRI, NHR, LHR, MHR, and PHR and the levels of neutrophils, monocytes, and triglycerides (TG) were higher in SCZ and BD groups, and levels of platelets, cholesterol (CHO), HDL, low-density lipoprotein (LDL), and apoprotein B (Apo B) were lower in SCZ and BD groups. Compared to the BD group, the values of the SIRI, lymphocytes, monocytes, and HDL were lower and the values of the SII, NHR, PHR, and platelet were higher in the SCZ group. In contrast to the BD-D group, the values of the SII; SIRI; NHR; and MHR; and levels of neutrophils, monocytes, and platelets were higher in the BD-M group, and the levels of CHO, TG, LDL, and Apo B were lower in the BD-M group. The MHR and NHR were predictors for differentiating the SCZ group from the HC group; the SIRI, NHR, and MHR were predictors for differentiating the BD-M group from the HC group; and the MHR was a predictor for differentiating the BD-D group from the HC group. The combination model of the indicators improved diagnostic effectiveness.

**Conclusion:**

Our study highlights the role of systemic inflammation in the pathophysiology of SCZ, BD-M, and BD-D, the association between inflammation and lipid metabolism, and these inflammation and lipid metabolism indicators showed different variation patterns in SCZ, BD-D, and BD-M.

## Introduction

Schizophrenia (SCZ) and bipolar disorder (BD) are two major psychiatric disorders, which lead to a decline in the social functioning of patients and impose a heavy burden on individuals, their families, and society, affecting a large number of people worldwide (1–4). Patients with SCZ have two to three times higher mortality than the general population and up to 15-year shorter life expectancy (5, 6). Mortality studies indicate that similar to SCZ, BDs are associated with a loss of approximately 10–20 potential years of life (1, 7). At present, the two common psychiatric illnesses are defined by clinical features in clinical practice, with considerable overlap in symptoms (3). However, they require different treatment strategies and have different treatment responses and outcomes. Features of BD and SCZ are partially overlapping, but valid and objective biomarkers for the diagnosis and differential diagnosis of these two diseases are still lacking. The pathogenesis of SCZ and BD has been studied by considerable research in the past. A meta-analysis showed that gray matter (GM) reductions in the anterior cingulate are markers of genetic liability to psychosis (8). Although significant progress in pathogenesis and treatment has been achieved, pathoetiology is based on several genetic and environmental factors, and there is not sufficient information explaining the etiology of SCZ and BD in the strict sense. In addition, there is still a subset of people who do not respond well to existing treatments, and some patients still have residual symptoms and functional impairment after undergoing treatment (9, 10). Therefore, the pathophysiological mechanisms of SCZ and BD need to be further studied, and the development of a valid method, exploring inexpensive and easily available biomarkers and resulting in an accurate diagnosis, is important and urgent.

A growing body of evidence suggests that immune dysfunction and inflammation have been considered to play important roles in the pathogenesis of SCZ and BD (11, 12). Many studies have shown that systemic low-grade inflammation, an attenuated but persistent form of the inflammatory response, is common in many psychiatric disorders including psychotic disorder, affective disorder, personality disorder, and neurotic disorder (13). Patients with SCZ and BD have been consistently shown to present with increased levels of pro-inflammatory cytokines, oxidative stress products, chemokines, and soluble adhesion molecules both in the peripheral blood and the cerebrospinal fluid (14, 15). A meta-analysis of cytokines in psychiatric disorders found that IL-6, TNF-α, IL-1RA, and sIL-2R were significantly elevated in peripheral blood of both acutely ill patients with SCZ and BD compared to those in healthy controls (16). Following treatment of the acute illness, IL-6 levels significantly decreased in SCZ, sIL-2R levels increased in SCZ, and IL-1RA levels decreased in bipolar mania (16).

Although there have been a number of studies linking inflammation to SCZ and BD, blood biomarker studies are the most frequently used method of study; however, many of these biomarkers are hard to routinely collect or are expensive to measure, which may limit its large-scale practical application in clinical practice. The levels of neutrophils, monocytes, lymphocytes, and platelets, which are inexpensive and can be easily acquired by performing a complete blood count test, change when there is inflammation in the organism (17). The systemic immune-inflammation index (SII) is a new marker reflecting the immune response and inflammation, defined as the platelet counts × neutrophil-to-lymphocyte ratio (18, 19). In some physical health disorders, such as ischemic stroke, cardiovascular disease, and pancreatitis, the SII has been found to be able to predict disease severity and prognosis (20–22). The system inflammation response index (SIRI) is an innovative inflammation-based biomarker, which integrates peripheral counts of neutrophils, monocytes, and lymphocytes (23); until now, the SIRI has been found to be a prognostic factor for cancers such as cholangiocarcinoma, nasopharyngeal carcinomas, and esophageal, gastric, and pancreatic cancers (24).

Furthermore, studies have found that high-density lipoprotein (HDL) showed antithrombotic, anti-inflammatory, and antioxidant effects, and HDL can inhibit the oxidation of low-density lipoprotein (LDL) and prevent its negative effects on the endothelium (25). In view of the role of neutrophils, monocytes, lymphocytes, platelets, and HDL in inflammation, the neutrophil/HDL ratio (NHR), lymphocyte/HDL ratio (LHR), monocyte/HDL ratio (MHR), and platelet/HDL ratio (PHR) have been recently investigated as new markers for inflammation, and they were suggested as potential markers of systemic inflammation and oxidative stress in many inflammatory diseases (26–30). They were found to be closely associated with the presence and prognosis of some physical health disorders, including cardiovascular disease, cancer, Parkinson’s disease, ischemic stroke, and erectile dysfunction (24, 27, 28, 31, 32). At present, there are few studies on these ratios in psychiatric diseases. A small-sample study on SCZ found that the MHR in patients with SCZ was significantly higher than that in healthy individuals (33).

To our knowledge, no large-scale study has evaluated the differences in the SII, SIRI, NHR, LHR, MHR, and PHR values in patients with SCZ and BD, and the relationship between these inflammation factors and SCZ and BD is not well understood. We hypothesized that these inflammatory indicators were associated with SCZ and BD, with varying levels. Therefore, the aim of this study was to use large-scale clinical data to evaluate whether these indicators were different among patients with SCZ, BD manic episodes (BD-M), BD depressive episodes (BD-D), and healthy individuals and whether these indicators would be able to act as potential biomarkers.

## Materials and methods

### Study population

A retrospective analysis was conducted on SCZ and BD patients hospitalized in the Beijing HuiLongGuan Hospital and Jining Psychiatric Hospital from January 2015 to January 2021. The sociodemographic and hematological data, extracted from the Electronic Medical Record System (EMRS) through a clinical record search tool, were evaluated in this cross-sectional, retrospective, naturalistic study. Only the first entry for each patient from the inpatient care unit was used for the analysis in this study. In general, the first blood test is executed on the next day after admission to the hospital. Therefore, the patients included in our study were in the acute phase of the disease.

Patients who met the following inclusion criteria were recruited: (a) patients with a diagnosis of SCZ or BD according to the International Statistical Classification of Diseases and Related Health Problems, 10th revision (ICD-10) diagnostic criteria; (b) those between 18 and 65 years at the time of blood analysis; and (c) those with available complete blood count parameters and lipid profile data. The criteria for exclusion were as follows: (a) significant acute or severe illnesses such as infection, autoimmune disease, heart failure, head injury, epilepsy, and tumor; (b) since significantly elevated or decreased white blood cells (WBC) and platelets indicate current infection, the subjects with leukocytosis (> 10 × 10^9^ cells/L), leukopenia (< 4 × 10^9^ cells/L), thrombocytosis (> 450 × 10^9^ cells/L), or thrombocytopenia (< 100 × 10^9^ cells/L) were also excluded to reduce the risk of including individuals affected by severe inflammatory diseases. A total of 13,329 patients with SCZ and 6,005 patients with BD who met the study criteria were included as the SCZ group and BD group, and the BD group contains 4,061 patients with BD manic episodes (BD-M) and 1,944 patients with BD depressive episodes (BD-D). For comparison, data from 5,810 healthy subjects with age and sex matched to the patient group were included as controls. The inclusion criteria of the healthy controls (HCs) were as follows: (a) those between 18 and 65 years at the time of blood analysis and (b) those with available complete blood count parameters and lipid profile data. The exclusion criteria of HCs were as follows: (a) patients with mental illnesses and significant acute or severe illnesses such as infection, autoimmune disease, heart failure, head injury, epilepsy, and tumor, and (b) those with leukocytosis, leukopenia, thrombocytosis, and thrombocytopenia.

### Demographic and clinical assessment

Basic demographic characteristics, including age and sex, were extracted from the EMRS. The data of complete blood count and lipid profile were also extracted from the EMRS, including the values of WBC, neutrophils, lymphocytes, monocytes, platelets, cholesterol (CHO), triglycerides (TG), HDL, LDL, and apoprotein B (Apo B). All subjects had their peripheral blood drawn in the morning (between 7 and 9 a.m.) on the day after admission to the hospital. Blood sample assays were conducted by laboratory technicians who were blinded to the patients’ diagnoses. The SII value was calculated as the ratio of neutrophil counts to lymphocyte counts multiplied by the platelet count (platelet × neutrophil-to-lymphocyte ratio). The SIRI value was calculated as the ratio of neutrophil counts to lymphocyte counts multiplied by the monocyte count (monocyte × neutrophil-to-lymphocyte ratio). The NHR value was calculated by dividing the neutrophil value by the HDL value (neutrophil/HDL), the LHR value was calculated by dividing the lymphocyte value by the HDL value (lymphocyte/HDL), the MHR value was calculated by dividing the monocyte value by the HDL value (monocyte/HDL), and the PHR was measured by dividing the platelet value by the HDL value (platelet/HDL).

### Ethical considerations

We informed the ethics committee of our hospital about this low-risk retrospective observational study, institutional ethical board approval was obtained, and a waiver of consent was provided.

### Statistical analysis

The statistical analysis of data obtained was analyzed using SPSS (statistical package for social sciences) 21.0. The normality of the data distributions was tested *via* the Kolmogorov–Smirnov test. The non-normal distribution data and the data of unequal variances were analyzed after log or sqrt transformation. Continuous data are described as mean ± standard error of mean (SEM). A *t*-test or one-way analysis of variance (ANOVA) was used. To exclude the confounding influence of age and sex, an analysis of covariance (ANCOVA) was employed, and a *post hoc* test using the Bonferroni correction analysis to correct for multiple comparisons was utilized to analyze the differences between groups. Chi-square (χ^2^) tests were employed for descriptive data. Spearman correlation analysis was used to analyze the relationships between neutrophil, lymphocyte, monocyte, platelet, and lipid profiles. A binary logistic regression analysis was performed to measure the impact of variables that were selected in univariate analysis by adjusting for covariates such as sex and age. A receiver operating characteristic (ROC) curve analysis was used to determine the factors affecting SCZ, BD-M, or BD-D and their diagnostic value. Considering the discriminatory capacity of the combinations of these measured indicators, we also analyzed the diagnostic value of the combined model calculated by binary logistic regression analysis. *P* < 0.05 was considered significant statistically in this analysis.

## Results

### Comparison of demographic features and laboratory indicators between schizophrenia, bipolar disorder, and healthy controls

In the present study, 19,334 patients with SCZ or BD and 5,810 healthy controls were enrolled for the final analysis. The mean age was 38.62 ± 0.090 years in the patient group, and 38.54 ± 0.145 years in the HC group. There were 9,425 males (48.7%) and 9,909 females (51.3%) in the patients group, and 2,850 males (49.1%) and 2,960 females (50.9%) in the HC group. There were no significant differences concerning age and sex within the patients group and HC group (*P* > 0.05). Further analysis of the demographic features among SCZ, BD, and HC groups found that there were no obvious differences concerning age among the three groups (*P* > 0.05), and the difference in terms of sex was statistically significant among the three groups (*P* < 0.01). SCZ, BD, and HC groups comprised 46.9%, 52.9%, and 49.1% male participants, respectively; the BD group had more male participants than SCZ and HC groups; and the SCZ group had more female participants than the HC group (*P* < 0.01).

**FIGURE 1:**
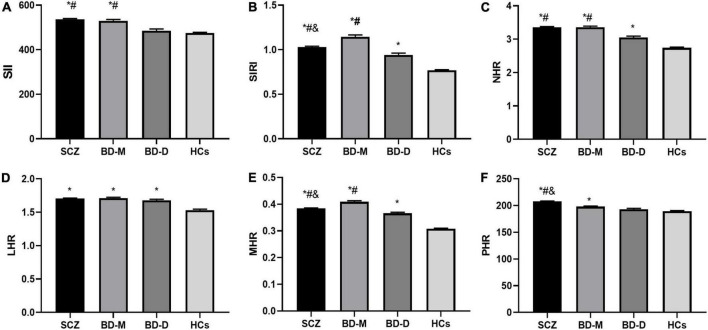
Comparison of inflammation ratios among SCZ, BD-M, BD-D, and HCs. **(A)** Comparison of SII; **(B)** comparison of SIRI; **(C)** comparison of NHR; **(D)** comparison of LHR; **(E)** comparison of MHR; **(F)** comparison of PHR. Data are represented as the mean ± SEM; * vs. HC group, *P* < 0.05; # vs. BD-D group, *P* < 0.05; & vs. BD-M group, *P* < 0.05. SCZ, schizophrenia; BD-M, bipolar disorder manic episodes; BD-D, bipolar disorder depressive episodes; HC, healthy control; SII, systemic immune-inflammation index; SIRI, system inflammation response index; NHR, neutrophil/HDL ratio; LHR, lymphocyte/HDL ratio; MHR, monocyte/HDLratio; PHR, platelet/HDLratio.

The ANCOVA showed that after adjusting for age and sex, there were significant differences in the values of SII, SIRI, MHR, NHR, LHR, and PHR and the levels of neutrophils, lymphocytes, monocytes, platelets, CHO, TG, HDL, LDL, and Apo B between SCZ, BD, and HC groups (*P* < 0.05). Further *post hoc* analyses showed that compared with the HC group, the values of the SII, SIRI, NHR, LHR, MHR, and PHR and the levels of neutrophils, monocytes, and TG were higher and levels of platelets, CHO, HDL, LDL, and Apo B were lower in SCZ and BD groups (*P* < 0.05). The SCZ group had lower lymphocyte counts than HC the group (*P* < 0.05), while no difference in lymphocyte counts was found between the BD and HC groups (*P* > 0.05). Compared to the BD group, the values of the SIRI, lymphocytes, monocytes, and HDL were lower and values of the SII, NHR, PHR, and platelets were higher in the SCZ group (*P* < 0.05), and there was no difference in values of the LHR, MHR, neutrophils, CHO, TG, LDL, and Apo B between SCZ and BD groups (*P* > 0.05). The variables of all participants and the results are shown in Figure 1 and Table 1.

**TABLE 1:** Comparison of demographic features and laboratory indicators between schizophrenia (SCZ), bipolar disorder (BD), and healthy controls (HCs).

Variables	SCZ group (*n* = 13329)	BD group (*n* = 6005)	HC group (*n* = 5810)	F/χ^2^	*P*
Age (year)	38.49 ± 0.106	38.89 ± 0.168	38.54 ± 0.145	2.249	0.106
Sex (male/female)	6247/7082^a^	3178/2827^ab^	2850/2960	60.930	0.000
SII	537.154 ± 3.312^ab^	515.046 ± 5.013^a^	475.470 ± 2.992	60.143	0.000
SIRI	1.030 ± 0.009^ab^	1.079 ± 0.016^a^	0.769 ± 0.006	186.345	0.000
NHR	3.362 ± 0.016^ab^	3.263 ± 0.024^a^	2.743 ± 0.019	284.742	0.000
LHR	1.705 ± 0.007^a^	1.701 ± 0.010^a^	1.530 ± 0.016	127.181	0.000
MHR	0.384 ± 0.002^a^	0.395 ± 0.003^a^	0.308 ± 0.002	407.848	0.000
PHR	207.829 ± 0.673^ab^	196.366 ± 0.983^a^	189.425 ± 1.086	129.572	0.000
Neutrophil	3.911 ± 0.014^a^	3.903 ± 0.023^a^	3.585 ± 0.015	96.305	0.000
Lymphocyte	1.981 ± 0.006^ab^	2.029 ± 0.009	2.014 ± 0.007	11.500	0.000
Monocyte	0.447 ± 0.001^ab^	0.472 ± 0.002^a^	0.402 ± 0.002	281.181	0.000
Platelet	244.808 ± 0.555^ab^	237.701 ± 0.830^a^	252.471 ± 0.744	75.406	0.000
CHO	4.321 ± 0.008^a^	4.326 ± 0.013^a^	4.908 ± 0.012	888.810	0.000
TG	1.412 ± 0.008^a^	1.441 ± 0.013^a^	1.302 ± 0.016	29.518	0.000
HDL	1.255 ± 0.003^ab^	1.292 ± 0.005^a^	1.446 ± 0.005	660.415	0.000
LDL	2.665 ± 0.007^a^	2.648 ± 0.011^a^	2.882 ± 0.010	175.775	0.000
Apo B	0.834 ± 0.002^a^	0.840 ± 0.003^a^	0.973 ± 0.014	32.264	0.000

SCZ, schizophrenia; BD, bipolar disorder; HC, healthy control; CHO, cholesterol; TG, triglycerides; HDL, high-density lipoprotein; LDL, low-density lipoprotein; Apo B, apoprotein B; SII, systemic immune-inflammation index; SIRI, system inflammation response index; NHR, neutrophil/HDL ratio; LHR, lymphocyte/HDL ratio; MHR, monocyte/HDL ratio; PHR, platelet/HDL ratio. a, vs. HC group, P < 0.05; b, vs. BD group, P < 0.05. Values are represented as mean ± SEM.

### Comparison between bipolar disorder manic episodes, bipolar disorder depressive episodes, and healthy controls

The patients with BD comprise 4,061 patients with BD manic episodes (BD-M) and 1,944 patients with BD depressive episodes (BD-D). There were no obvious differences in age among BD-M, BD-D, and HC groups (*P* > 0.05). There were more male participants in the BD-M group than in the HC group (*P* < 0.01), and no significant difference in sex was found between the BD-M and BD-D groups (*P* > 0.05).

The ANCOVA revealed statistically significant differences in the values of the SII, SIRI, NHR, LHR, MHR, and PHR and the levels of neutrophils, monocytes, platelets, CHO, TG, HDL, LDL, and Apo B among BD-M, BD-D, and HC groups (*P* < 0.05). In contrast to the HC group, both the BD-M and BD-D groups showed higher SIRI, NHR, LHR, MHR, monocytes, and TG and lower platelets, CHO, HDL, LDL, and Apo B (*P* < 0.05). Furthermore, the BD-M group displayed higher SII, PHR, and neutrophils than the HC group (*P* < 0.05), whereas the SII, PHR, and neutrophils showed no difference between the BD-D and HC groups (*P* > 0.05). In contrast to the BD-D group, the values of SII, SIRI, MHR, and NHR and levels of neutrophils, monocytes, and platelets were higher in BD-M, and the levels of CHO, TG, LDL, and Apo B were lower in BD-M (*P* < 0.05), whereas there were no differences concerning LHR, PHR, and HDL within the two groups (*P* > 0.05). The results are shown in Figure 1 and Table 2.

**TABLE 2:** Comparison of demographic features and laboratory indicators between BD manic episodes (BD-M), BD depressive episodes (BD-D), and healthy controls (HCs).

Variables	BD-M group (*n* = 4061)	BD-D group (*n* = 1944)	HC group (*n* = 5810)	F/χ^2^	*P*
Age (year)	38.81 ± 0.202	39.05 ± 0.302	38.54 ± 0.145	1.503	0.222
Sex (male/female)	2176/1885^a^	1002/942	2850/2960	19.819	0.000
SII	529.547 ± 6.234^ab^	484.754 ± 8.340	475.470 ± 2.992	36.500	0.000
SIRI	1.145 ± 0.021^ab^	0.942 ± 0.020^a^	0.769 ± 0.006	185.605	0.000
NHR	3.362 ± 0.031^ab^	3.056 ± 0.036^a^	2.743 ± 0.019	155.548	0.000
LHR	1.712 ± 0.013^a^	1.677 ± 0.017^a^	1.530 ± 0.016	68.999	0.000
MHR	0.409 ± 0.004^ab^	0.366 ± 0.004^a^	0.308 ± 0.002	300.570	0.000
PHR	197.926 ± 1.222^a^	193.107 ± 1.642	189.425 ± 1.086	11.472	0.000
Neutrophil	4.012 ± 0.028^ab^	3.675 ± 0.036	3.585 ± 0.015	95.784	0.000
Lymphocyte	2.039 ± 0.010	2.008 ± 0.015	2.014 ± 0.007	2.085	0.124
Monocyte	0.487 ± 0.003^ab^	0.440 ± 0.004^a^	0.402 ± 0.002	340.330	0.000
Platelet	239.398 ± 1.011^ab^	234.155 ± 1.449^a^	252.471 ± 0.744	85.704	0.000
CHO	4.261 ± 0.015^ab^	4.462 ± 0.024^a^	4.908 ± 0.012	617.065	0.000
TG	1.403 ± 0.016^ab^	1.521 ± 0.025^a^	1.302 ± 0.016	25.116	0.000
HDL	1.295 ± 0.006^a^	1.286 ± 0.008^a^	1.446 ± 0.005	238.927	0.000
LDL	2.588 ± 0.013^ab^	2.774 ± 0.020^a^	2.882 ± 0.010	174.155	0.000
Apo B	0.823 ± 0.004^ab^	0.874 ± 0.006^a^	0.973 ± 0.014	52.797	0.000

BD-M, bipolar disorder manic episodes; BD-D, bipolar disorder depressive episodes; HC, healthy controls; CHO, cholesterol; TG, triglycerides; HDL, high-density lipoprotein; LDL, low-density lipoprotein; Apo B, apoprotein B; SII, systemic immune-inflammation index; SIRI, system inflammation response index; NHR, neutrophil/HDL ratio; LHR, lymphocyte/HDL ratio; MHR, monocyte/HDL ratio; PHR, platelet/HDL ratio. a, vs. HC group, P < 0.05; b, vs. BD-D group, P < 0.05. Values are represented as mean ± SEM.

### Comparison between schizophrenia, bipolar disorder manic episodes, and bipolar disorder depressive episodes

A comparison of age among SCZ, BD-M, and BD-D groups presented that no difference was noted (*P* > 0.05). Chi-square analysis showed that both the BD-M and BD-D groups had an increased male/female ratio compared with the SCZ group (*P* < 0.01).

After adjusting for age and sex, the ANCOVA showed that the differences in the values of SII, SIRI, NHR, MHR, and PHR and the levels of neutrophils, lymphocytes, monocytes, platelets, CHO, TG, HDL, LDL, and Apo B among the three groups were statistically significant (*P* < 0.05). When compared with the BD-D group, the SCZ group showed higher SII, SIRI, NHR, MHR, PHR, neutrophils, monocytes, and platelets but lower CHO, TG, HDL, LDL, and Apo B than the BD-D group (*P* < 0.05), while there was no difference in lymphocyte counts between the two groups (*P* > 0.05). In comparison with the BD-M group, the SCZ group presented higher PHR, platelet, CHO, LDL, and Apo B but lower SIRI, MHR, neutrophil, lymphocyte, monocyte, and HDL than the BD-M group (*P* < 0.05), whereas no significant differences were found with regard to SII, NHR, and TG between the two groups (*P* > 0.05). Among the three groups, the BD-M group showed the highest SIRI, MHR, neutrophils, and monocytes but the lowest CHO, LDL, and Apo B; the BD-D group showed the lowest SIRI, MHR, neutrophils, monocytes, and platelets but highest CHO, LDL, and Apo B; and the SCZ group showed highest PHR and platelets but lowest HDL. The data are shown in Figure 1 and Table 3.

**TABLE 3:** Comparison of demographic features and laboratory indicators between BD manic episodes (BD-M), BD depressive episodes (BD-D), and healthy controls (HCs).

Variables	SCZ group (*n* = 13329)	BD-M group (*n* = 4061)	BD-D group (*n* = 1944)	F/χ^2^	*P*
Age (year)	38.49 ± 0.106	38.81 ± 0.202	39.05 ± 0.302	2.287	0.102
Sex (male/female)	6247/7082^ab^	2176/1885	1002/942	62.936	0.000
SII	537.154 ± 3.312^a^	529.547 ± 6.234^a^	484.754 ± 8.340	15.006	0.000
SIRI	1.030 ± 0.009^ab^	1.145 ± 0.021^a^	0.942 ± 0.020	24.661	0.000
NHR	3.362 ± 0.016^a^	3.362 ± 0.031^a^	3.056 ± 0.036	29.918	0.000
LHR	1.705 ± 0.007	1.712 ± 0.013	1.677 ± 0.017	2.251	0.103
MHR	0.384 ± 0.002^ab^	0.409 ± 0.004^a^	0.366 ± 0.004	30.072	0.000
PHR	207.829 ± 0.673^ab^	197.926 ± 1.222	193.107 ± 1.642	47.458	0.000
Neutrophil	3.911 ± 0.014^ab^	4.012 ± 0.028^a^	3.675 ± 0.036	26.168	0.000
Lymphocyte	1.981 ± 0.006^b^	2.039 ± 0.010	2.008 ± 0.015	10.781	0.000
Monocyte	0.447 ± 0.001^ab^	0.487 ± 0.003^a^	0.440 ± 0.004	76.340	0.000
Platelet	244.808 ± 0.555^ab^	239.398 ± 1.011^a^	234.155 ± 1.449	20.934	0.000
CHO	4.321 ± 0.008^ab^	4.261 ± 0.015^a^	4.462 ± 0.024	28.200	0.000
TG	1.412 ± 0.008^a^	1.403 ± 0.016^a^	1.521 ± 0.025	10.195	0.000
HDL	1.255 ± 0.003^ab^	1.295 ± 0.006	1.286 ± 0.008	37.639	0.000
LDL	2.665 ± 0.007^ab^	2.588 ± 0.013^a^	2.774 ± 0.020	34.960	0.000
Apo B	0.834 ± 0.002^ab^	0.823 ± 0.004^a^	0.874 ± 0.006	28.342	0.000

SCZ, schizophrenia; BD-M, bipolar disorder manic episodes; BD-D, bipolar disorder depressive episodes; CHO, cholesterol; TG, triglycerides; HDL, high-density lipoprotein; LDL, low-density lipoprotein; Apo B, apoprotein B; SII, systemic immune-inflammation index; SIRI, system inflammation response index; NHR, neutrophil/HDL ratio; LHR, lymphocyte/HDL ratio; MHR, monocyte/HDL ratio; PHR, platelet/HDL ratio. a, vs. BD-D group, P < 0.05; b, vs. BD-M group, P < 0.05. Values are represented as mean ± SEM.

### Correlations between neutrophil, lymphocyte, monocyte, platelet, and lipid profiles

As shown in Table 4, the correlations between neutrophil, lymphocyte, monocyte, platelet, and lipid profiles were analyzed by Spearman correlation analysis in the groups. In the HC group, the neutrophil count was positively correlated with CHO, TG, and LDL (*P* < 0.01) and negatively correlated with HDL (*P* < 0.01). The lymphocyte count showed positive correlations with CHO, TG, LDL, and Apo B (*P* < 0.01), while showed a negative correlation with HDL (*P* < 0.01). The monocyte count displayed positive correlations with CHO, TG, LDL, and Apo B (*P* < 0.05), while displaying a negative correlation with HDL (*P* < 0.01). The platelet count was positively correlated with CHO, TG, and LDL (*P* < 0.01).

**TABLE 4:** Correlations among neutrophil, lymphocyte, monocyte, platelet, and lipid profile indicators.

Parameters	Neutrophil	Lymphocyte	Monocyte	Platelet
**HC Group**				
CHO	0.046**	0.131**	0.058**	0.133**
TG	0.264**	0.232**	0.242**	0.061**
HDL	−0.238**	−0.186**	−0.245**	–0.008
LDL	0.084**	0.151**	0.095**	0.123**
Apo B	0.102	0.181**	0.143*	0.105
**SCZ Group**				
CHO	0.078**	0.096**	0.049**	0.172**
TG	0.176**	0.243**	0.110**	0.093**
HDL	−0.103**	−0.149**	−0.097**	0.039**
LDL	0.088**	0.103**	0.082**	0.177**
Apo B	0.107**	0.085**	0.120**	0.183**
**BD-M Group**				
CHO	0.065**	0.061**	0.039*	0.109**
TG	0.108**	0.204**	0.102**	0.027
HDL	−0.076**	−0.126**	−0.085**	0.081**
LDL	0.086**	0.088**	0.063**	0.125**
Apo B	0.112**	0.076**	0.111**	0.131**
**BD-D Group**				
CHO	0.100**	0.055*	0.031	0.146**
TG	0.128**	0.136**	0.069**	0.051*
HDL	−0.050*	−0.116**	−0.061**	0.082**
LDL	0.117**	0.054*	0.044	0.153**
Apo B	0.144**	0.038	0.070**	0.157**

Results given as Spearman correlation coefficient. HCs, healthy controls; SCZ, schizophrenia; BD-M, bipolar disorder manic episodes; BD-D, bipolar disorder depressive episodes; CHO, cholesterol; TG, triglycerides; HDL, high-density lipoprotein; LDL, low-density lipoprotein; Apo B, apoprotein B. *, P < 0.05; **, P < 0.01.

In the SCZ group, the neutrophil, lymphocyte, monocyte, and platelet counts were all positively correlated with CHO, TG, LDL, and Apo B (*P* < 0.01); neutrophil, lymphocyte, and monocyte counts were all negatively correlated with HDL (*P* < 0.01), while the platelet count was positively correlated with HDL (*P* < 0.01).

In the BD-M group, neutrophils, lymphocytes, and monocytes all showed positive correlations with CHO, TG, LDL, and Apo B (*P* < 0.05), while showed a negative correlation with HDL (*P* < 0.01). The platelet count was positively correlated with CHO, HDL, LDL, and Apo B (*P* < 0.01).

In the BD-D group, neutrophils and platelets were all positively correlated with CHO, TG, LDL, and Apo B (*P* < 0.05), the neutrophil count was negatively correlated with HDL (*P* < 0.05), while the platelet count was positively correlated with HDL (*P* < 0.01). The lymphocyte count had positive correlations with CHO, TG, and LDL (*P* < 0.05), while had a negative correlation with HDL (*P* < 0.01). The monocyte had positive correlations with TG and Apo B (*P* < 0.01), while had a negative correlation with HDL (*P* < 0.01).

### The influencing factors for the occurrence of schizophrenia, bipolar disorder manic episodes, or bipolar disorder depressive episodes

To analyze the relationship between the inflammation ratios including SII, SIRI, NHR, LHR, MHR, and PHR and the disease status, multivariable logistic regression analysis was performed using variables that were selected in univariate analysis to identify the factors associated with the occurrence of SCZ, BD-M, or BD-D by adjusting for covariates such as sex and age. In binary logistic regression analysis, the disease status was used as the dependent variable, and age, sex, SII, SIRI, NHR, LHR, MHR, and PHR were used as covariates.

**FIGURE 2:**
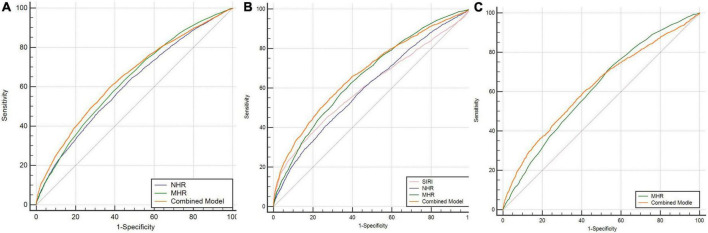
**(A)** ROC curves for the diagnostic ability of the parameters for SCZ (SCZ vs. HCs). NHR: AUC 0.611 (95% CI = 0.604 to 0.618), *P* < 0.001; MHR: AUC 0.632 (95% CI = 0.625 to 0.639), *P* < 0.001; combined model: AUC 0.647 (95% CI = 0.639 to 0.655), *P* < 0.001. **(B)** ROC curves for the diagnostic ability of the parameters for BD-M (BD-M vs. HCs). SIRI: AUC 0.608 (95% CI = 0.598 to 0.618), *P* < 0.001; NHR: AUC 0.603 (95% CI = 0.593 to 0.612), *P* < 0.001; MHR: AUC 0.661 (95% CI = 0.651 to 0.670), *P* < 0.001; combined model: AUC 0.677 (95% CI = 0.668 to 0.686), *P* < 0.001. **(C)** ROC curves for the diagnostic ability of the parameters for BD-D (BD-D vs. HCs). MHR: AUC 0.615 (95% CI = 0.606 to 0.626), *P* < 0.001; combined model: AUC 0.623 (95% CI = 0.612 to 0.634), *P* < 0.001. SCZ, schizophrenia; BD-M, bipolar disorder manic episodes; BD-D, bipolar disorder depressive episodes; HCs: healthy controls.

In the analysis of the SCZ, as shown in Table 5, the binary logistic regression model included SII, SIRI, NHR, MHR, PHR, and sex, they were independently associated with the occurrence of SCZ. In the multivariate analysis, the SIRI, NHR, MHR, and PHR were positively associated with SCZ, while the SII was inversely associated with SCZ. According to the ROC curves, the parameters with an area under curve (AUC) higher than 0.6 were MHR [AUC 0.632 (0.625–0.639), *P* = 0.000, cutoff 0.29, sensitivity 63.01%, specificity 55.49%] and NHR [AUC 0.611 (0.604–0.618), *P* = 0.000, cutoff 2.47, sensitivity 64.78%, specificity 51.05%], and the AUCs of SII, SIRI, and PHR were lower than 0.6. The combination model of the indicators improved diagnostic effectiveness, with an AUC value of 0.647 [AUC 0.647 (0.639–0.655), *P* = 0.000, cutoff 0.68, sensitivity 59.48%, specificity 62.79%]. The data are shown in Figure 2A.

**TABLE 5:** Binary logistic regression analysis results.

Variables	SCZ			BD-M			BD-D		
	OR	*95%CI*	*p*	OR	*95%CI*	*p*	OR	*95%CI*	*p*
Age	–	–	–	–	–	–	1.005	1.000–1.009	0.044
Sex	0.636	0.594–0.682	0.000	0.760	0.694–0.834	0.000	0.843	0.751–0.946	0.004
SII	0.915	0.888–0.944	0.000	0.941	0.898–0.986	0.010	–	–	–
SIRI	1.937	1.622–2.312	0.000	2.852	2.208–3.684	0.000	1.997	1.661–2.401	0.000
NHR	1.068	1.025–1.112	0.002	0.850	0.796–0.907	0.000	0.873	0.803–0.949	0.000
LHR	–	–	–	1.436	1.251–1.649	0.000	1.522	1.296–1.787	0.000
MHR	4.337	2.588–7.270	0.000	9.775	4.734–20.186	0.000	4.384	2.267–8.477	0.000
PHR	1.176	1.073–1.290	0.001	0.813	0.712–0.928	0.002	–	–	–

SCZ, schizophrenia; BD-M, bipolar disorder manic episodes; BD-D, bipolar disorder depressive episodes; SII, systemic immune-inflammation index; SIRI, system inflammation response index; NHR, neutrophil/HDL ratio; LHR, lymphocyte/HDL ratio; MHR, monocyte/HDL ratio; PHR, platelet/HDL ratio.

In the binary logistic regression analysis of BD-M, the SII, SIRI, NHR, LHR, MHR, and PHR, and demographic features, such as sex, were included in the logistic model, which was independent indicators of BD-M. In the multivariate analysis, the SIRI, LHR, and MHR were positive predictors of BD-M, while the SII, NHR, and PHR were inversely associated with BD-M (data are shown in Table 5). The ROC curve analysis of BD-M, the indicators with an AUC higher than 0.6 were the SIRI [AUC 0.608 (0.598–0.618), *P* = 0.000, cutoff 0.91, sensitivity 44.67%, specificity 73.43%], NHR [AUC 0.603 (0.593–0.612), *P* = 0.000, cutoff 3.3, sensitivity 41.05%, specificity 73.39%], and MHR [AUC 0.661 (0.651–0.670), *P* = 0.000, cutoff 0.31, sensitivity 62.23%, specificity 60.93%]; the AUCs of the SII, LHR, and PHR were lower than 0.6. The AUC value of the combination model of the indicators was 0.677 [AUC 0.677 (0.668–0.686), *P* = 0.000, cutoff 0.41, sensitivity 54.91%, specificity 71.34%]. The data are shown in Figure 2B.

The binary logistic regression analysis in BD-D showed that SIRI, NHR, LHR, MHR, sex, and age were included in the logistic model, and they were independent influencing factors for the occurrence of BD-D. The SIRI, LHR, and MHR were positively correlated with BD-D, while the NHR was inversely associated with BD-D in the multivariate analysis (data are shown in Table 5). In the ROC curve analysis of BD-D, the parameters with an AUC higher than 0.6 were the MHR [AUC 0.615 (0.604–0.626), *P* = 0.000, cutoff 0.26, sensitivity 70.78%, specificity 46.83%], and the AUCs of the SII, SIRI, NHR, LHR, and PHR were lower than 0.6. The AUC value of the combination model of the indicators was 0.623 [AUC 0.623 (0.612–0.634), *P* = 0.000, cutoff 0.24, sensitivity 59.57%, specificity 59.21%]. The data are shown in Figure 2C.

## Discussion

This study suggested that systemic inflammation occurs in SCZ and BD, and SCZ, BD-M, and BD-D had different inflammation indicators and variation patterns. To the best of our knowledge, this is the first large-scale study to compare multiple common hematological parameters in terms of inflammation, lipid metabolism, and their integration indicators including SII, SIRI, NHR, LHR, MHR, and PHR among Chinese patients with SCZ, BD-M, and BD-D.

The results of the present study revealed that both the patients with SCZ and BD had higher values of SII, SIRI, NHR, LHR, MHR, PHR, neutrophils, monocytes, and TG and lower levels of platelets, CHO, HDL, LDL, and Apo B than HCs. Compared to the BD group, the values of the SIRI, lymphocytes, monocytes, and HDL were lower in the SCZ group, and values of SII, NHR, PHR, and platelets were higher in the SCZ group. Further analysis displayed that in contrast to the BD-D group, BD-M had higher values of SII, SIRI, MHR, NHR, neutrophil, monocyte, platelet, and lower levels of CHO, TG, LDL, and Apo B. In the SCZ, BD-M, and BD-D groups, the BD-M group showed the highest SIRI, MHR, neutrophils, and monocytes but the lowest CHO, LDL, and Apo B; the BD-D group showed the lowest SIRI, MHR, neutrophils, monocytes, and platelets but highest CHO, LDL, and Apo B; the SCZ group showed highest PHR and platelets but lowest HDL. The observation also showed that lipid profile HDL exhibited negative correlations with neutrophils, monocytes, and lymphocytes in SCZ, BD-M, and BD-D. The MHR and NHR were predictors for differentiating SCZ patients from HCs; the SIRI, NHR, and MHR were predictors for differentiating BD-M patients from HCs; the MHR was the predictor for differentiating BD-D patients from HCs. There are different molecular patterns in the SCZ, BD-M, and BD-D, and the combination model of the indicators improved diagnostic effectiveness.

A growing number of studies have explored the role of inflammation in psychiatric disorders, with many suggesting that inflammation is involved in the pathogenesis of SCZ and BD (33). New inflammatory markers with the characteristics of convenience, non-invasive nature, low price, and reproducibility have been a focus of researchers. The SII, SIRI, MHR, NHR, LHR, and PHR are considered cost-effective and simple laboratory parameters indicating systemic inflammation in many diseases (18, 19, 21–23, 26, 27, 30–34). They can reveal the immune response balance, and in many inflammatory diseases, including cancers, coronary heart disease, and coronavirus disease 2019 (COVID-19), they are considered independent predictors (24, 35–37). In the present study, we found that patients with SCZ and BD had higher values of SII, SIRI, MHR, NHR, LHR, and PHR than HCs, and the results indicated that systemic inflammation occurs in SCZ and BD.

The NHR, LHR, MHR, and PHR are the integrative indicators of systemic inflammation which combined HDL and complete blood count test indicators including neutrophils, lymphocytes, monocytes, and platelets. Neutrophils, lymphocytes, monocytes, platelets, and HDL are all closely associated with inflammation and oxidative stress (38, 39). In the present study, we found that all SCZ, BD-M, and BD-D groups had a higher monocyte level and a lower platelet level than HCs, and the SCZ and BD-M groups had a higher neutrophil level, while the SCZ group showed a lower lymphocyte count than HCs. In the SCZ, BD-M, and BD-D groups, the BD-M group showed the highest neutrophil and monocyte levels, the SCZ group showed the highest platelet level, while the BD-D group showed the lowest neutrophil, monocyte, and platelet levels. Neutrophils are the most abundant type of white blood cells in the body, the key cell types in the innate immune system, and the first line of cellular defense against infection (40). Lymphocytes are components of the adaptive immune system and play a major role in the body’s immune response, including antibody production and cell-mediated immunity (41). Monocytes, important components of the innate immune response against pathogens, represent the most important cells for the secretion of pro-inflammatory and pro-oxidant cytokines (42, 43). Platelets can regulate the permeability of endothelial cells and the recruitment of neutrophils, macrophages, and their effectors, and activated platelets have inflammatory functions in several physiological and pathological conditions (44, 45). All of them are easily accessible with complete blood count test parameters. HDL, a component of lipid profile, can prevent the accumulation of free CHO and TG in the vessels; protect endothelial cells against adverse effects of LDL; and prevent oxidation of LDL (46). HDL also has pleiotropic protective functions, including anti-infectious, anti-inflammatory, antioxidant, anti-thrombotic, anti-inflammatory, and immunomodulatory properties (39, 47). Our study showed that HDL had an association with the neutrophils, lymphocytes, monocytes, and platelets and these had also been verified in previous research. HDL has been found to inhibit the activation, attachment, diffusion, and migration of neutrophils (43) and the activities of monocytes (48). Furthermore, HDL can inhibit antigen presentation-mediated T-cell activation by disrupting lipid rafts in antigen-presenting cells (49); they also play a role in the prevention of platelet hyperreactivity (50). As for the complex interactions among neutrophils, lymphocytes, monocytes, platelets, and HDL, the combined indicator including NHR, LHR, MHR, and PHR might be more reliable in reflecting the inflammation level than a single parameter.

Our results showed that compared to the HCs, the patients with SCZ, BD-M, and BD-D had higher NHR, LHR, and MHR; SCZ and BD-M groups displayed higher PHR; whereas the BD-D group showed no difference in PHR compared with the HC group. In the three groups of SCZ, BD-M, and BD-D, both SCZ and BD-M patients showed higher NHR than BD-D patients, while no difference was found in NHR between the SCZ and BD-M groups. In the three groups, the patients with BD-M showed the highest MHR, followed by SCZ; the BD-D group showed the lowest MHR; the SCZ group showed the highest PHR, followed by BD-M and BD-D. There is no difference in the LHR between the three groups. The results suggested the elevation of the inflammation level in SCZ and BD, especially in BD manic episodes and SCZ. The binary logistic regression analysis in the present study found that the values of the NHR, MHR, and PHR were independently correlated with SCZ, while the NHR, LHR, MHR, and PHR were independently associated with BD-M, as well as NHR, LHR, and MHR were independently correlated with BD-D. ROC curve analysis showed that the MHR and NHR were independent predictors for differentiating SCZ or BD-M patients from HCs, and the AUC of the MHR was higher than that of the NHR; the MHR was an independent predictor for differentiating BD-D patients from HCs, and the AUC of the combined model was higher than that of the single indicator. These findings suggested that patients with SCZ, BD manic episodes, and BD depressive episodes had different variation patterns of inflammatory indicators: the MHR had a higher predictive value for differentiating SCZ, BD-M, or BD-D patients from HCs than other indicators. Multiple inflammation indicators are more promising predictors than any single indicator considered in isolation. Previous studies also indicated that patients with schizophrenia had higher MHR values than HCs, and the MHR had a positive correlation with the severity of the disease (33).

The SII, which brings together three inflammatory peripheral cell counts including neutrophils, lymphocytes, and platelets, is increasingly considered a readily available biomarker for systemic inflammation (20, 35, 36). It can better reflect the body’s immune and inflammatory states than any aforementioned markers in isolation. Previous studies have found that the SII has an important role in predicting the prognosis of patients with some physical diseases, such as tumors, cerebral infarction, cardiovascular disease, and acute pancreatitis (18, 20, 21, 36). As far as we know, there are few studies on the SII in psychiatric disorders. Our results showed that compared to HCs, the SCZ and BD patients had a higher SII. Further analysis found that SCZ and BD-M patients showed a higher SII than BD-D patients and HCs, while SCZ patients had no difference in the SII compared with BD-M patients, and BD-D patients had no difference compared with HCs. Binary logistic regression analysis in the present study showed that the SII was independently correlated with SCZ and BD-M. The results suggested that the SII value may be increased in SCZ and the mania state of BD but not in the depression state of BD. The SIRI is a new inflammation-based biomarker integrating peripheral counts of neutrophils, lymphocytes, and monocytes (23). In a previous study, the SIRI was found to reflect the inflammation response and have a prognosis value in many cancers, including cholangiocarcinoma, and esophageal, and gastric cancers (24). To the best of our knowledge, the SIRI had not been studied previously in patients with SCZ and BD. Our study found that patients with SCZ, BD-M, and BD-D had a higher SIRI than HCs; patients with BD-M had the highest SIRI, followed by patients with SCZ and then BD-D. The SIRI was an independent influencing factor for the occurrence of SCZ, BD-M, and BD-D. Our results also indicated that a high SIRI was an independent predictor for differentiating patients with BD-M from HCs. These results suggest a more significant association between SIRI with BD mania episodes. Our results suggest the role of the SIRI in SCZ and BD, especially in BD manic episodes.

Another important finding of the study was the alteration of lipid metabolism in SCZ and BD. Our results discovered that SCZ, BD-M, and BD-D groups had higher TG and lower CHO, HDL, LDL, and Apo B than HCs. In the SCZ, BD-M, and BD-D groups, the BD-D group showed the highest CHO, LDL, and Apo B, followed by the SCZ group; the BD-M group showed the lowest CHO, LDL, and Apo B; furthermore, the SCZ group showed the lowest HDL. Several reports had identified lower cholesterol levels in BD manic patients than in the euthymic phase or BD depressive patients (51), which was in line with our results. Cholesterol is a principal component of the cell membrane and plays an important role in synaptic functions, and the depletion of cholesterol may induce the alteration of brain signaling, with effects on several neurotransmission systems (48, 52). In line with our results, a lower HDL cholesterol level had been reported in SCZ and BD patients in previous studies. Some research studies have found that reduced HDL levels were associated with smaller hippocampal volume and cognitive deficits in BD patients (53). Lower levels of HDL cholesterol are correlated with an increased vulnerability to oxidative damage to lipids, proteins, and DNA, and increased immune–inflammatory responses (53). Furthermore, our results showed that lipid profiles had correlations with neutrophils, lymphocytes, monocytes, and platelets, suggesting a link between lipid metabolism and inflammation.

There are some strengths and limitations in the present study. First, the study analyzed the inflammation ratios of 13,329 patients with SCZ and 6,005 patients with BD, and 5,810 healthy subjects. To the best of our knowledge, this is the largest study that explored peripheral inflammation ratios in SCZ and BD. Furthermore, the real-world measures in the present study increased the clinical transferability of the results. In addition, the study compared the SII, SIRI, NHR, LHR, MHR, and PHR among SCZ, BD-M, and BD-D patients. To our knowledge, this is the first study in this area. However, some limitations must be acknowledged. First, the retrospective design did not allow us to conduct a structured psychiatric interview and assessment of the severity of symptoms, so the association between inflammation ratios and severity of symptoms was not analyzed in the present study. Second, the effects of treatment were not accounted for in this study, which may affect the levels of the indicators. Third, some confounders, such as smoking, diet, and body mass index, may influence the inflammation ratios, and due to the limitations of the feasibility of this study, we were not able to control some factors. Fourth, there are many other promising biomarkers in terms of inflammation that are not included in our study. In the future, more specific potential biomarkers should be explored. Finally, our research was a retrospective, cross-sectional study, the direction of the relationship between inflammation ratios and the onset of SCZ and BD was not allowed for causal inference. Longitudinal studies are needed to investigate the interaction between these inflammation ratios and SCZ and BD over time.

## Conclusion

In conclusion, this study demonstrated that SCZ, BD-M, and BD-D have unique variation patterns of inflammation ratios and lipid metabolism indicators, and highlighted the role of systemic inflammation in the pathophysiology of SCZ, BD-M, and BD-D, and the association between inflammation and lipid metabolism. This research demonstrated that there were significant differences in the inflammation indicators including SII, SIRI, NHR, LHR, MHR, and PHR among patients with SCZ, BD-M, and BD-D. In the three groups, the BD-M group showed the highest SIRI, MHR, neutrophils, and monocytes but the lowest CHO, LDL, and Apo B; the BD-D group showed the lowest SIRI, MHR, neutrophil, monocyte, and platelet but the highest CHO, LDL, and Apo B; and the SCZ group showed the highest PHR and platelets but the lowest HDL. The observation also showed that the lipid profiles had correlations with neutrophils, lymphocytes, monocytes, and platelets, suggesting a link between lipid metabolism and inflammation. The MHR and NHR were predictors for differentiating SCZ patients from HCs; the SIRI, NHR, and MHR were predictors for differentiating BD-M patients from HCs; and the MHR was a predictor for differentiating BD-D patients from HCs. The combination model of the indicators improved diagnostic effectiveness. These indicators may be peripheral trait biomarkers, which may reflect enhanced inflammatory signaling in SCZ, BD-M, and BD-D. In the future, these indicators may be used to assist in the identification and differential diagnosis of SCZ and BD. The present study could serve as a key reference for future research, and more solid links between these inflammation ratios and psychiatric diseases may be discovered in further longitudinal studies.

## Data availability statement

The raw data supporting the conclusions of this article will be made available by the authors, without undue reservation.

## Ethics statement

The studies involving human participants were reviewed and approved by the ethics committee of Beijing HuiLongGuan Hospital. Written informed consent for participation was not required for this study in accordance with the national legislation and the institutional requirements.

## Author contributions

YW designed the study, wrote the main manuscript, and prepared the tables and figures. TW, GL, JF, and LD collected and analyzed the data. HX and LY reviewed and edited the manuscript. JM and DC collected the data. JC designed the study and supervised the entire process. All authors reviewed the manuscript.

## Conflict of interest

The authors declare that the research was conducted in the absence of any commercial or financial relationships that could be construed as a potential conflict of interest.

## Publisher’s note

All claims expressed in this article are solely those of the authors and do not necessarily represent those of their affiliated organizations, or those of the publisher, the editors and the reviewers. Any product that may be evaluated in this article, or claim that may be made by its manufacturer, is not guaranteed or endorsed by the publisher.
